# Employing advanced supervised machine learning approaches for predicting micronutrient intake status among children aged 6–23 months in Ethiopia

**DOI:** 10.3389/fnut.2024.1397399

**Published:** 2024-06-11

**Authors:** Alemu Birara Zemariam, Molalign Aligaz Adisu, Aklilu Abera Habesse, Biruk Beletew Abate, Molla Azmeraw Bizuayehu, Wubet Tazeb Wondie, Addis Wondmagegn Alamaw, Habtamu Setegn Ngusie

**Affiliations:** ^1^Department of Pediatrics and Child Health Nursing, School of Nursing, College of Medicine and Health Science, Woldia University, Woldia, Ethiopia; ^2^Department of Pediatrics and Child Health Nursing, School of Nursing, College of Medicine and Health Science, Ambo University, Ambo, Ethiopia; ^3^Department of Emergency and Critical Care Nursing, School of Nursing, College of Medicine and Health Science, Woldia University, Woldia, Ethiopia; ^4^Department of Health Informatics, School of Public Health, College of Medicine and Health Science, Woldia University, Woldia, Ethiopia

**Keywords:** micronutrient supplementation, children, machine learning algorithm, prediction, Ethiopia

## Abstract

**Background:**

Although micronutrients (MNs) are important for children’s growth and development, their intake has not received enough attention. MN deficiency is a significant public health problem, especially in developing countries like Ethiopia. However, there is a lack of empirical evidence using advanced statistical methods, such as machine learning. Therefore, this study aimed to use advanced supervised algorithms to predict the micronutrient intake status in Ethiopian children aged 6–23 months.

**Methods:**

A total weighted of 2,499 children aged 6–23 months from the Ethiopia Demographic and Health Survey 2016 data set were utilized. The data underwent preprocessing, with 80% of the observations used for training and 20% for testing the model. Twelve machine learning algorithms were employed. To select best predictive model, their performance was assessed using different evaluation metrics in Python software. The Boruta algorithm was used to select the most relevant features. Besides, seven data balancing techniques and three hyper parameter tuning methods were employed. To determine the association between independent and targeted feature, association rule mining was conducted using the *a priori* algorithm in R software.

**Results:**

According to the 2016 Ethiopia Demographic and Health Survey, out of 2,499 weighted children aged 12–23 months, 1,728 (69.15%) had MN intake. The random forest, catboost, and light gradient boosting algorithm outperformed in predicting MN intake status among all selected classifiers. Region, wealth index, place of delivery, mothers’ occupation, child age, fathers’ educational status, desire for more children, access to media exposure, religion, residence, and antenatal care (ANC) follow-up were the top attributes to predict MN intake. Association rule mining was identified the top seven best rules that most frequently associated with MN intake among children aged 6–23 months in Ethiopia.

**Conclusion:**

The random forest, catboost, and light gradient boosting algorithm achieved a highest performance and identifying the relevant predictors of MN intake. Therefore, policymakers and healthcare providers can develop targeted interventions to enhance the uptake of micronutrient supplementation among children. Customizing strategies based on identified association rules has the potential to improve child health outcomes and decrease the impact of micronutrient deficiencies in Ethiopia.

## Introduction

Micronutrient intake is the provision of either a single micronutrient (MN), such as iodine, zinc, calcium, manganese, chromium, copper, fluoride, iron, folic acid, vitamin A, vitamin B-complex, or vitamin D, or a combination of MNs. These essential nutrients can be administered in the form of capsules, tablets, drops, or syrups (1). MNs are essential and required in small amounts. However, when there is a deficiency in their supply, it can have significant negative effects on the growth and development of children. These effects include stunting, wasting, delayed cognitive development, prolonged hospital stays, and weakened immunity, making children more susceptible to common childhood infections (2, 3). Despite the crucial role that MNs play in promoting healthy growth and development in children, there has been limited focus on ensuring an adequate intake of these nutrients (4). MN deficiency continues to be a widespread public health issue, particularly in developing countries like Ethiopia (5).

Due to the latency nature of clinical symptoms of MN deficiency as far as they are not detected via blood levels in an early stages with some exceptions, inadequacy of one or more of its supplementation leads to health consequence or hidden hunger (6). The deficiency of MNs, combined with stunting and wasting, contributes to approximately 45% or 3.1 million deaths in children every year (7). As per the 2019 report from the United Nations Children’s Fund, approximately 340 million children globally, with 54% of them residing in developing countries, experienced hidden hunger due to deficiencies in micronutrients (8).

To address MN deficiency and its consequences, different countries have implemented MN supplementation or improved intake strategies. Scientific evidences have shown that providing high doses of MNs like vitamin A, iron, and zinc can lead to a reduction of childhood mortality (9). Despite the efforts of programs such as the WHO 2016–2025 nutrition strategy, the inadequate intake of MNs remains a persistent issue in both developed and developing countries (10). For example, in Brazil, only 54.2% of children aged 6–59 months consumed a micronutrient supplement (11), and less than 10% of children aged 6–59 months in Ethiopia received iron supplements and deworming tablets (10, 12).

A systematic review covering Ethiopia, Nigeria, Kenya, and South Africa found varying rates of micronutrient (MN) intake, ranging from 51 to 99% for zinc, 13 to 100% for iron, and 1 to 100% for vitamin A (13). The consumption of iodized salt varied from 2% in Kenya to 96% in Ethiopia (14). In another study involving children aged 6–23 months across 20 sub-Saharan countries, it was reported that nearly 74% of the children had adequate micronutrient intake, with Ethiopian children having the lowest intake at round of to 59% (15).

The Ethiopian government has made significant efforts to address national nutrition issues, including the implementation of the first national nutrition program in 2008 (16), which prioritized ending malnutrition. It also joined the Scaling up Nutrition movement in 2012 and the Seqota Declaration in 2015 to combat child undernutrition by 2030 (17). However, despite these efforts, the issue of malnutrition and the deficiency of MN intake remain significant public health concerns in Ethiopia. According to the 2016 Ethiopian Demographic and Health Survey (EDHS), only 14% of children aged 6–23 months consumed minimum dietary diversity (12).

Various factors, including maternal socio-demographics, child characteristics, and maternal healthcare services utilization, are associated with MN intake (1, 14, 15, 18, 19). Previous studies in Ethiopia have used classical statistical methods to analyze MN intake status (14, 15), which means that the estimates are based on the previous assumptions, which may limit the potential to discover hidden information and these strategies are used to analyze features selected based on prior knowledge or logical reasoning and it is difficult to handle complex data patterns and capturing nonlinear relationships, which are often present in dietary intake data. Leveraging machine learning (ML) models can offer significant advantages and contribute to the existing empirical evidence and making the most accurate predictions enabling systems to learn from data rather than making prior assumptions (20). ML techniques excel in managing complex and nonlinear data, operate without preexisting assumptions, and capture intricate relationships among predictors (20, 21). Besides, the previous study was confined with a limited ML algorithm, data balancing techniques, and small sample size. Therefore, this study aimed to utilize 12 advanced ML techniques including association rule mining to predict MN intake status and identify its predictors using the 2016 EDHS data set. The findings will inform policymakers in planning evidence-based programs with integrated interventions to enhance MN intake. Moreover, these findings can provide valuable insights for developing context-specific strategies to address these issues, inform targeted interventions, policy-making, and resource allocation aimed at improving the nutritional status of children in Ethiopia, ultimately contributing to enhanced public health outcomes.

## Methods

### Study settings, data sources, and sampling procedures

Data from the 2016 Ethiopian Demographic and Health Surveys (EDHS) were obtained through a formal written request to the DHS program website (22).^1^ The DHS Program has conducted standardized surveys in over 90 countries, gathering comprehensive and representative data on aspects such as population, health, HIV, and nutrition. The EDHS data includes information from nine regions [Tigray, Afar, Amhara, Oromia, Benishangul-Gumuz, Gambela, South Nation Nationalities and Peoples’ Region (SNNPR), Harari, and Somali] as well as two administrative cities (Addis Ababa and Dire-Dawa). The data collection procedure utilized a multi-stage stratified cluster sampling approach for each region. Stratification was performed based on urban and rural sectors, and enumeration areas were selected using probability proportional to size. Within the chosen enumeration areas, households were selected using equal probability systematic sampling (23). The study focused on children aged 6–23 months in Ethiopia within the previous 5 years. The analysis involved a weighted sample size of 2,499 children aged 6–23 months. The dataset employed in the study included 23 distinct features that were considered during the analysis.

### Study variables and measurements

The study variable of interest is the micronutrient intake status (MNs) among children aged 6–23 months. To determine the MN intake status, we have considered six options: consumption of food rich in vitamin A (VA) or iron within the past 24 h, consumption of micronutrient powders (MNP) or iron supplements within the past 7 days, and receipt of vitamin A supplementation (VAS) or deworming treatment within the past 6 months (24–26). To determine the intake of the minimum recommended MNs, if the respondent reported that the child had consumed at least one of the minimum recommended MNs, it was classified as a “Yes” response. Conversely, if the child had not received any of the recommended MNs, it was classified as a “No” response.

To assess the consumption of foods rich in vitamin A (VA), we analyzed the intake of seven specific food groups within the previous 24 h. These food groups included eggs, various meats (such as beef, pork, lamb, and chicken), pumpkin, carrots, and squash, dark green leafy vegetables, mangoes, papayas, and other fruits rich in VA, as well as liver, heart, and other organs, and fish or shellfish. Similarly, we assessed the consumption of iron-rich foods by examining the intake of four specific food groups within the previous 24 h. These food groups consisted of eggs, various meats, liver, heart, and other organs, as well as fish or shellfish. To determine the intake of MNP, we asked the respondents if their child had received such powders in the past 7 days. For assessing iron supplementation, we inquired whether the child had been given iron pills, sprinkles with iron, or iron syrup within the past 7 days. The researchers examined vitamin A supplementation (VAS) and deworming treatment by reviewing the integrated child health card, which contains information on immunization and growth monitoring history. They also obtained verbal responses from the mothers. These assessments were specifically conducted for children aged 6–23 months to determine if they had received VAS and deworming treatment in the last 6 months. If the respondent reported that the child had consumed at least one of these food groups, it was categorized as a “Yes” response, indicating the consumption of MN-rich foods.

The study considered several independent variables, including place of residence, region, religion, media exposure, sex of household head, age of mother, age of child, ANC visit, postnatal care (PNC) visit, family size, current marital status, working status of the mother, desire for more children, current pregnancy status, number of children, place of delivery, mode of delivery, history of diarrhea, history of cough, sex of child, working status of the father, educational status of the mother, educational status of the father, and wealth index. The selection of these independent variables was based on a comprehensive review of existing literature in the field.

### Data preprocessing

**Figure 1:**
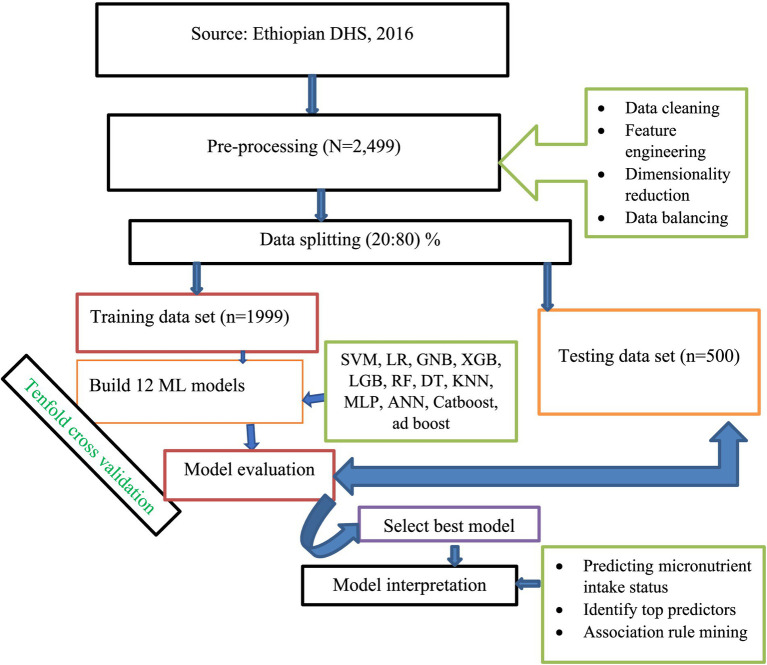
Study workflow diagram.

The first step in ML is data pre-processing, which involves modifying or encoding the data to make it understandable by computers (27). In our ML workflow, we employed a process of continuous improvement for our models. This process included selecting and engineering features, choosing models, and tuning hyper-parameters. We refined our models continuously over an iterative approach. Figure 1 provides the details of the specific steps in our workflow.

### Data cleaning

We performed a thorough examination to identify and eliminate any duplicated data entries in our dataset. After this review, we verified that there were no redundant entries present. To address missing values, we applied the K-nearest neighbors (KNN) imputation technique (28). To detect outliers, we utilized different methods such as box plots and Grubbs’ test. Furthermore, we evaluated multicollinearity by examining the correlation matrix. We considered a correlation value above 0.8 between two variable pairs as an indication of high correlation (29, 30).

#### Feature engineering

Feature engineering involves identifying, obtaining, and adjusting the most important features from the available data to develop accurate and efficient ML models (31). In our study, we employed one-hot encoding to encode nominal categorical variables and label encoding for ordinal categorical variables (32).

#### Dimensionality reduction

We did feature selection to enhance the model performance and reduce the dimensionality of the dataset (33). We utilized a feature selection method called Boruta, which assesses the importance of features by comparing their performance with randomly generated shadow features that mimic noise. Features that consistently outperformed the shadow features were considered significant and included in our predictive model (34).

#### Data balancing

Data mining and ML face difficulties due to class imbalance, which leads to reduced accuracy and biased estimate when classifying minority instances (35). To address this challenge, it is advisable to explore different data balancing techniques and select the one that performs well, as the effectiveness of these techniques can vary depending on the nature of the dataset. To mitigate this issue, we utilized seven data balancing techniques, including under-sampling, over-sampling, adaptive synthetic sampling (ADASYN), (SMOTE), synthetic minority oversampling technique with edited nearest neighbors (SMOTE ENN), SMOTE Tomik, and the near miss methods. Initially, we trained our ML algorithms using the unbalanced data. Then, we investigated the mentioned balancing techniques to train the models using balanced data. To assess the performance of each model, we compared accuracy, AUC (Area under the Curve), and other evaluation metrics. It is recommended to consider both accuracy and AUC, along with other pertinent metrics, to thoroughly evaluate model performance and make informed comparisons between various ML algorithms (36–38). Taking these factors into account, we have chosen the balancing technique that exhibited superior performance for further tuning and the final prediction of the micronutrient supplementation status.

### Model selection and development

In our study, the variable of interest, which indicated the status of micronutrient intake, necessitated a classification method as it was divided into two distinct categories: “yes” and “no.” To make accurate predictions, it was essential to choose suitable classifiers. To achieve this, we utilized the scikit-learn version 1.3.2 libraries in Python, implemented through Jupyter Notebook, to apply a range of ML algorithms.

To assess the predictive capabilities of ML algorithms in predicting the status of micronutrient supplementation, we employed 12 advanced machine learning algorithms. These algorithms encompassed support vector machines with kernel methods, Gaussian naive Bayes, logistic regression, decision tree classifier, random forest classifier, gradient boosting machines, extreme gradient boosting, AdaBoost Classifier, k-nearest neighbors, CatBoost Classifier, MLP Classifier, and ANN with tensor flow.

### Model training and evaluation

Creating a dependable predictive model in ML entails essential steps in model training and evaluation (39, 40). In our study, we adopted a straightforward approach by splitting the data into an 80% training set and a 20% testing set. To assess the performance of each predictive model, we employed various evaluation metrics, including accuracy, precision, recall, F1-score, and AUC. Accuracy gaged overall correctness, precision measured accurate positive predictions, recall evaluated the identification of all positive instances, and the F1-score provided a balanced measure. AUC, calculated from the area under the ROC curve, indicated the algorithm’s ability to discriminate between classes (41).

To further evaluate the model’s performance, we employed a 10-fold cross-validation techniques (28). Additionally, we conducted a comprehensive analysis of hyper parameters to refine and enhance the model’s performance. It is recommended to experiment with various tuning techniques and take the one which perform better from the others. Accordingly, we systematically explored grid search, random search, and Bayesian optimization. By comparing the results from these techniques, we identified configurations that yielded the highest performance. To improve the accuracy and reliability of the model, we also performed model calibration. Through fine-tuning the model via calibration, we enhanced its predictive capabilities to accurately forecast the desired outcome. Various kernel methods were also compared for SVM model.

### Model interpretability

Incorporating SHAP (SHapley Additive exPlanations) values and association rule mining has been highlighted by scholars for achieving diverse objectives (42, 43). Association rule mining is suitable for uncovering hidden patterns and relationships within the data, while SHAP analysis is more appropriate for understanding the impact of different features on the overall model predictions (42, 44).

Therefore, we have utilized a variety of techniques. Initially, we computed the mean SHAP values to evaluate the average impact of each feature on the model’s predictions, providing insights into the relative significance of different variables. Subsequently, we employed a waterfall plot to visually depict the cumulative effects of these variables, emphasizing their contributions to the overall prediction. Lastly, association rule mining was employed to uncover concealed patterns and relationships among the variables, enabling a more profound exploration of the dataset.

## Results

### Descriptive results of the participants characteristics

A total weighted sample of 2,499 children aged 6–23 months was included in this study. Among these children, 1,728 (69.15%) had a micronutrient intake. Approximately two-thirds (63.99%) of the participants fell within the age range of 12 to 23 months, and more than half (59.38%) of their mothers did not have any formal education. In terms of wealth status and religion, 50.5% of the respondents belonged to the poor wealth quintile, and roughly 49.74% identified as Muslim. A majority (69.07%) of the respondents had a history of antenatal care (ANC) visits, while the majority (91.04%) had not received any PNC service. Additionally, more than three-quarters (79.35%) of the respondents resided in rural areas, and nearly two-thirds (64.71%) had no access to media exposure. The detailed statistics are presented in Table 1.

**Table 1:** Individual characteristics of respondents in Ethiopia, 2016 (*N* = 2,499).

Variable	Categories	Frequency	Percent
Mothers age in years	15–24	739	29.57
	25–34	1,283	51.34
	> = 35	477	19.09
Residence	Urban	516	20.65
	Rural	1983	79.35
Religion	Muslim	1,243	49.74
	Orthodox	742	29.69
	Protestant	454	18.17
	Others*	60	2.4
Mothers educational status	No education	1,484	59.38
	Primary education	700	28.01
	Secondary education	199	7.96
	Higher education	116	4.64
Region	Tigray	267	10.68
	Afar	234	9.36
	Amhara	226	9.04
	Oromia	360	14.41
	Somali	301	12.04
	Benishangule gumez	204	8.16
	SNNP	326	13.05
	Gambella	160	6.4
	Harari	153	6.12
	Addis Abeba	132	5.28
	Diredawa	136	5.44
Wealth index	Poor	1,262	50.5
	Medium	370	14.81
	Rich	867	34.69
Desire for more children	Wants	1737	69.51
	Undecided	110	4.4
	No more wants	652	26.09
Fathers educational status	No formal education	1,111	44.46
	Primary education	855	34.21
	Secondary education	307	12.28
	Higher education	226	9.04
Fathers occupational status	Not working	247	9.88
	Working	2,252	92.12
Mothers occupational status	Not working	1,527	61.1
	Working	972	38.9
Child age in months	6–11	900	36.01
	12–23	1,599	63.99
ANC follow-up	Yes	1726	69.07
	No	773	30.93
Place of delivery	Home	1,433	57.34
	Health facility	1,066	42.66
PNC follow-up	Yes	224	8.96
	No	2,275	91.04
Media exposure	Yes	882	35.29
	No	1,617	64.71

Others*, catholic, traditional; ANC, antenatal care; PNC, post-natal care.

### Machine learning approaches of micronutrient intake status

#### Feature selection

**Figure 2:**
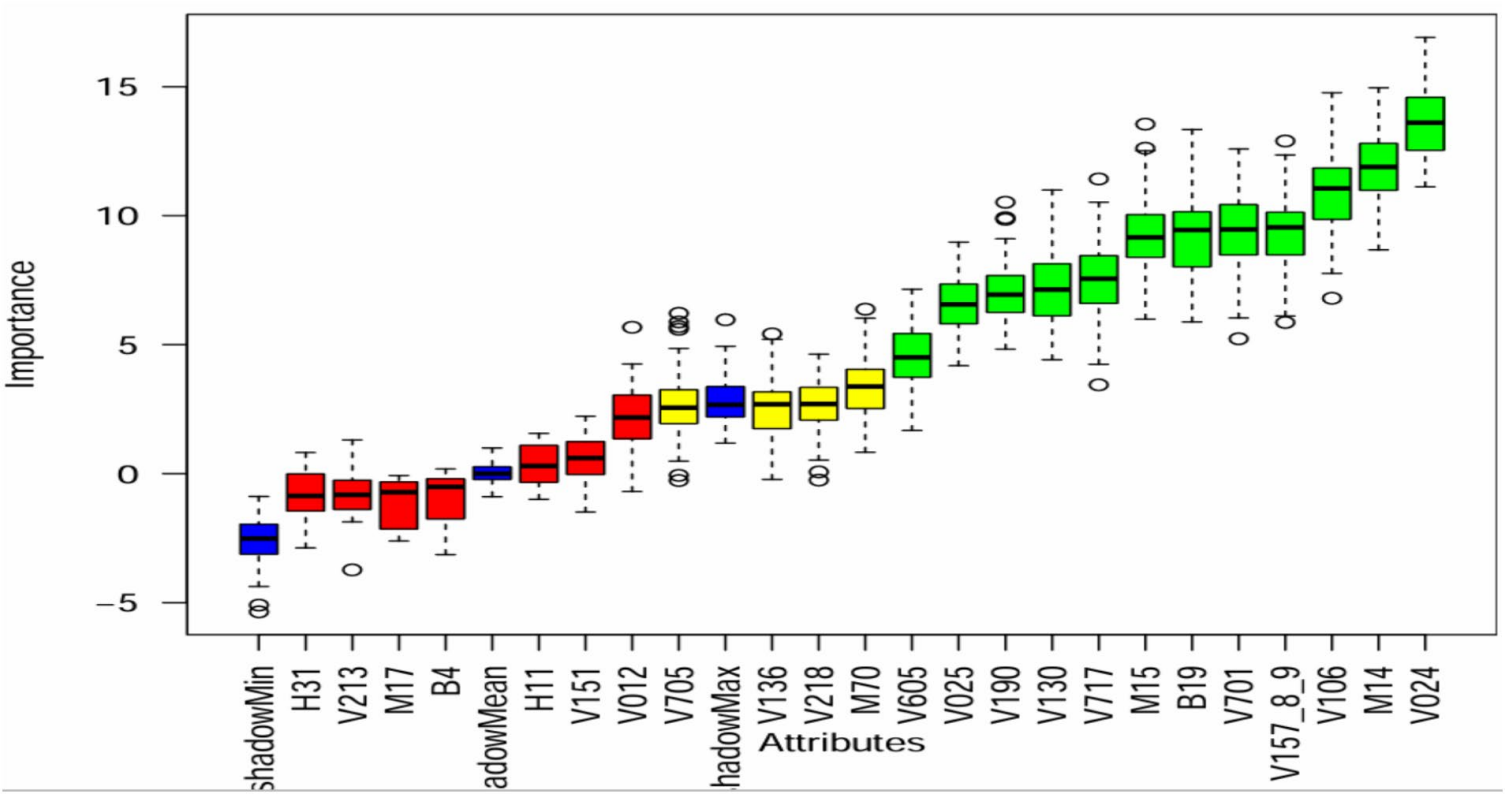
Feature selection using Boruta algorithm H31-had cough, V213-current pregnancy status, M17-Mode of delivery, B4-sex of child, H11-had diarrhea, V151-sex of household head, V012-mothers age, V705-husband occupation, V136-family size, V218-number of living children, M70-PNC visit, V605-desire more children, V025-residence, V190-wealth index, V130-religion, V717-mother occupation, M15-place of delivery, B19-age of child, V701-husband education status, V157_8_9-media exposure status, V106-educational status of respondent, M14-ANC visit, and V024-region.

Upon evaluating different methods for feature selection, we found that the Boruta algorithm produced favorable outcomes. The graphical representation of the Boruta algorithm effectively illustrated the significance of various variables, with significant variables displayed in green, insignificant variables in red, and uncertain variables in yellow (45). Our analysis of the Boruta algorithm graph (Figure 2) revealed that seven variables were considered insignificant or unimportant, four variables were uncertain, and the remaining 12 variables were deemed important for predicting the status of micronutrient intake. Consequently, we employed 16 variables to forecast micronutrient intake and explore data patterns using association rule mining.

### Data balancing

Table 2 provides a comparison of various data balancing techniques, including under-sampling, over-sampling, Adaptive Synthetic Sampling (ADASYN), synthetic minority over-sampling technique (SMOTE), synthetic minority over-sampling technique with edited nearest neighbors (SMOTE ENN), SMOTE tomek, and the near-miss algorithm. Among these techniques, SMOTE ENN demonstrated the highest performance, achieving an AUC above 0.90 for all ML algorithms except Gaussian naive Bayes, logistic regression, and the decision tree classifier. Notably, Gaussian naive Bayes achieved an AUC of 0.85, logistic regression achieved an AUC of 0.89, and the decision tree classifier achieved an AUC of 0.84, all of which were considered acceptable. Additionally, the SMOTE ENN data balancing technique achieved an accuracy value above 85.0% across all 12 ML algorithms. These findings indicate that SMOTE ENN outperformed than the other data balancing techniques, as shown in Table 2. Consequently, we have selected SMOTE ENN as the final and most effective data balancing technique for further ML analysis and optimization. For a comprehensive visual representation comparing the performance of ML algorithms with each data balancing technique, please refer to Supplementary Figure 1.

**Table 2:** Comparisons of different imbalanced data handling techniques.

Algorithm	Parameters	Unbalanced data	Under-sampling	Over-sampling	ADASYN	SMOTE	SMOTE ENN	SMOTE TOMEK	Near miss
SVM	Accuracy	72	70	73	73	77	91	71	65
	AUC	0.67	0.70	0.73	0.73	0.77	0.91	0.78	0.65
GNB	Accuracy	62	69	68	70	74	85	65	64
	AUC	0.67	0.69	0.68	0.70	0.74	0.85	0.74	0.64
LR	Accuracy	70	68	68	71	73	89	68	64
	AUC	0.68	0.68	0.68	0.71	0.73	0.89	0.75	0.64
DT	Accuracy	62	59	67	65	68	84	70	54
	AUC	0.58	0.59	0.67	0.65	0.68	0.84	0.68	0.54
RF	Accuracy	71	69	80	76	80	97	75	59
	AUC	0.66	0.69	0.80	0.76	0.80	0.97	0.81	0.59
LGB	Accuracy	71	71	75	74	78	94	70	62
	AUC	0.69	0.71	0.75	0.74	0.78	0.94	0.79	0.62
XGB	Accuracy	69	66	76	72	77	94	73	60
	AUC	0.66	0.66	0.76	0.72	0.77	0.94	0.78	0.60
KNN	Accuracy	71	69	68	71	74	90	69	61
	AUC	0.67	0.69	0.68	0.71	0.74	0.90	0.75	0.61
MLP	Accuracy	67	64	72	71	74	93	71	57
	AUC	0.66	0.64	0.72	0.71	0.74	0.93	0.75	0.57
Adaboost	Accuracy	72	73	71	74	76	92	70	65
	AUC	0.71	0.73	0.71	0.74	0.76	0.92	0.78	0.65
Catboost	Accuracy	72	71	77	75	79	96	73	65
	AUC	0.69	0.71	0.77	0.75	0.79	0.96	0.80	0.65
ANN	Accuracy	69	68	73	72	75	93	70	59
	AUC	0.67	0.68	0.73	0.72	0.75	0.93	0.75	0.59

AUC, area under curve; ANN, artificial neural network; ADASYN, Adaptive Synthetic Sampling; DT, decision tree; MLP, multilayer perceptron; KNN, k nearest neighboring; XGB, extreme gradient boosting; LGB, light gradient boosting; RF, random forest; SMOTE, Synthetic Minority Over-sampling Technique; ENN, Edited Nearest Neighbors; GNB, Gaussian naïve Bayes; SVM, support vector machine; LR, logistic regression.

### Development and performance comparisons of ML-based models

During the analysis, we evaluated multiple ML algorithms to predict the status of micronutrient intake. To assess the algorithms’ performance, we considered various metrics such as accuracy, precision, sensitivity, specificity, recall, F1 score, and AUC. Additionally, we employed techniques like grid search, random search, and Bayesian optimization for fine-tuning the models to improve prediction accuracy. The detail of evaluation metrics is presented in Supplementary Figure 2.

The results demonstrated that all 12 algorithms performed exceptionally well, although their specific performance varied depending on the tuning technique used. When employing the grid search technique, the random forest, catboost, and light gradient boosting algorithm achieved the highest performance with an AUC of 0.98. Most algorithms achieved an AUC above 0.90, except for Gaussian naive Bayes, logistic regression, and decision trees, which had AUC values of 0.82, 0.85, and 0.87, respectively.

In the random search hyper parameter tuning, the random forest, light gradient boosting, and catboost algorithms demonstrated equally impressive performance metrics, achieving an AUC of 0.94. Similar to the results obtained from the grid search technique, most algorithms achieved an AUC above 0.90, except for Gaussian naive Bayes and decision trees, which had AUC values of 0.88. When employing the Bayesian optimization technique, the catboost algorithm followed by MLP achieved superior AUC values of 0.98 and 0.96, respectively.

In general, the comprehensive evaluation revealed excellent performance across all 12 ML algorithms, with consistent and comparable results. Different tuning techniques yielded the best outcomes for different algorithms, with random search, grid search, and Bayesian optimization demonstrating notable performance in specific cases. While some variations in performance were observed, no single technique consistently outperformed all aspects of the ML algorithms.

The random forest, light gradient boosting, and catboost algorithms with grid search and random search optimization tuning emerged as the top three performers across all metrics. In addition, the catboost algorithm followed by MLP exhibited strong performance when tuned with Bayesian optimization.

**Figure 3:**
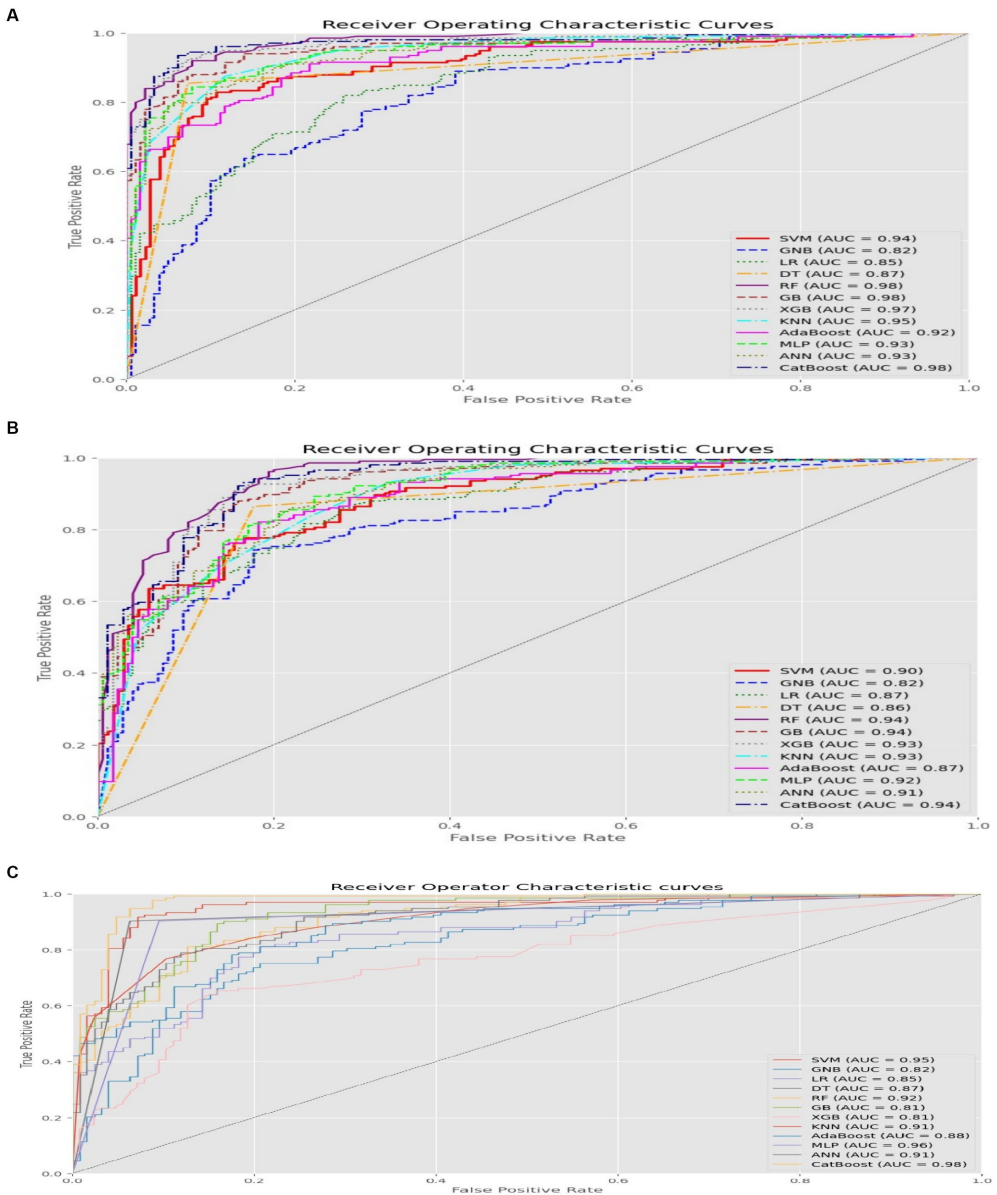
AUC value of each ML algorithm. **(A)** Grid search, **(B)** Random search, **(C)** Bayesian optimization.

For a comprehensive comparison of the 12 ML algorithms and their performance across the three tuning techniques, please refer to Table 3. Furthermore, graphical representations illustrating the performance of each algorithm under different tuning techniques can be found in Figure 3.

**Table 3:** Accuracy and AUC value of ML algorithms using three hyper parameter tuning techniques.

Algorithm	Grid search		Random search		Bayesian optimization	
	Accuracy	AUC	Accuracy	AUC	Accuracy	AUC
SVM	94	0.94	85	0.90	92	0.95
GNB	82	0.82	73	0.82	72	0.82
LR	85	0.85	79	0.87	78	0.85
DT	87	0.87	82	0.86	82	0.87
RF	98	0.98	87	0.94	83	0.97
LGB	98	0.98	87	0.94	68	0.81
XGB	97	0.97	87	0.93	68	0.81
KNN	95	0.95	85	0.93	82	0.91
MLP	93	0.93	87	0.92	90	0.96
Adaboost	92	0.92	78	0.87	80	0.88
Catboost	98	0.98	89	0.94	94	0.98
ANN	93	0.93	86	0.91	81	0.91

### Model interpretability

#### SHAP value interpretation

**Figure 4:**
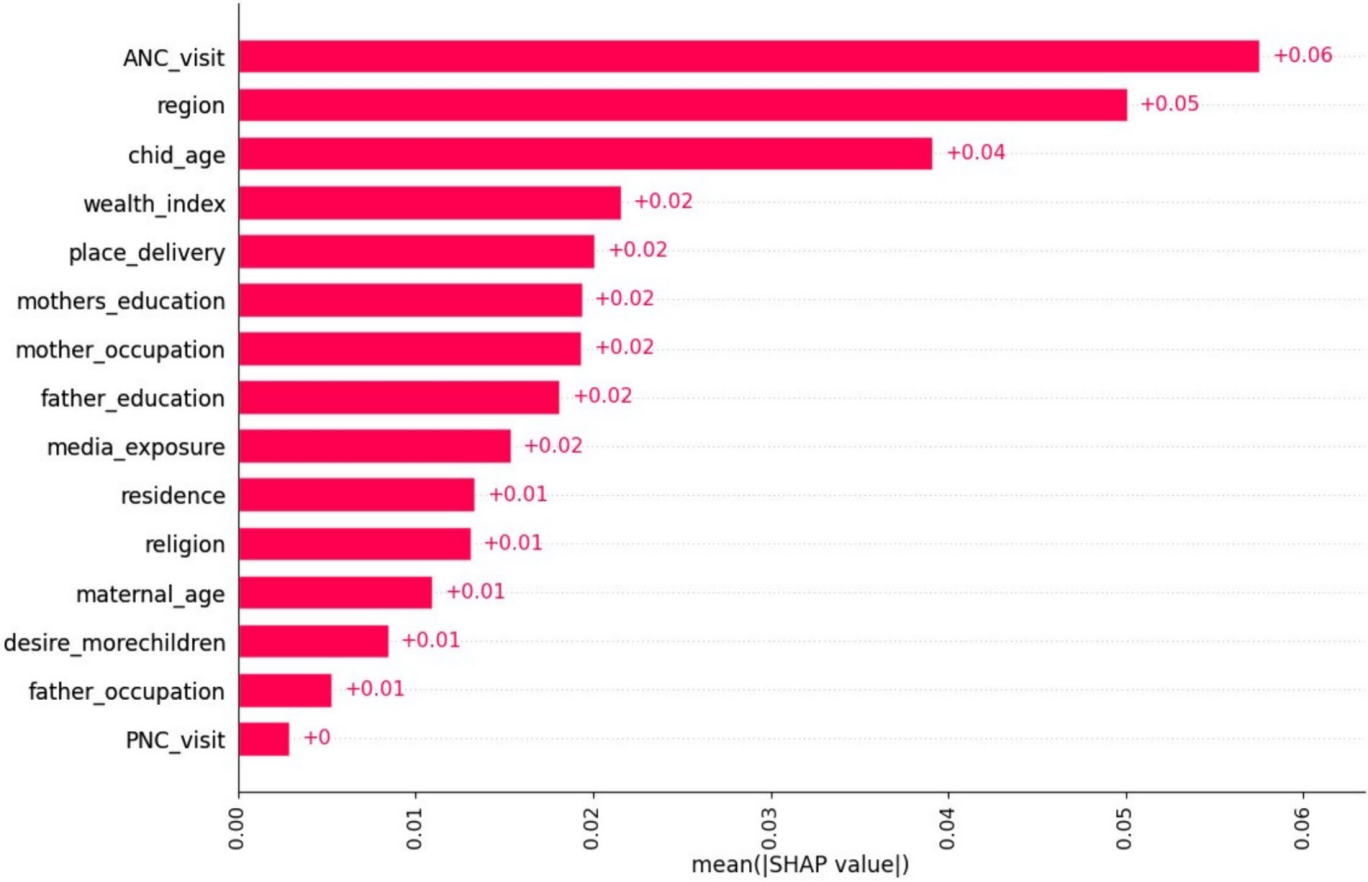
A mean SHAP value report.

Based on the information presented in Figure 4, the mean SHAP value report provided valuable insights into the relative importance of different features in the classification model. Factors such as ANC visit, region, and child age emerged as the most influential variables, exerting a significant impact on the model’s predictions. This suggests that these features play a crucial role in determining the model’s predictions, while the remaining six variables have minimal influence.

**Figure 5:**
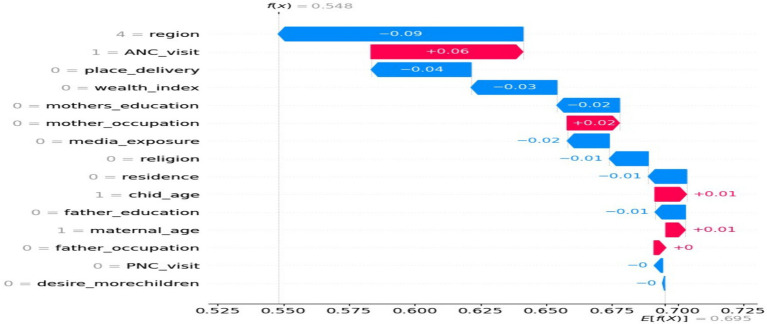
Waterfall plot.

The findings depicted in Figure 5, as shown by the waterfall plot, offer valuable insights into the hierarchy of feature importance for predicting the target variable. The plot highlights that ANC visit has the highest positive impact on the prediction, followed by mother occupation, child age, and mother age. On the other hand, place of delivery, wealth index, mother education, and media exposure negatively contribute to the model’s prediction. This indicates that factors such as home delivery, poor wealth status, lack of formal education, and absence of media exposure are associated with lower predicted outcomes in the model, while their absence or opposite attributes are associated with higher predicted outcomes.

### Association rule mining

By utilizing the *a priori* algorithm, we were able to identify significant association rules with a confidence level exceeding 95%. These rules provided valuable insights into the likelihood of micronutrient intake status among children aged 6–23 months in Ethiopia. Notably, certain variables such as region, wealth index, place of delivery, mothers’ occupation, child age, fathers’ educational status, desire for more children, access to media exposure, and ANC follow-up consistently appeared in these rules, indicating their strong association with the probability of micronutrient intake. In total, 167 rules were generated, and the following are the top seven association rules ranked by their confidence levels and corresponding lift values.

1. If children living in Benishangul Gumez region and children from medium wealth index household, the probability of micronutrient intake status is 98.7% (lift value = 1.45)
2. If children living in Gambella region, children from rich household, and whose mothers gave birth at health facility, the probability of micronutrient intake is 97.8% (lift value = 1.43)
3. If children living in Benishangul Gumuz region, children from rich household, and children who had a mother with work or occupation, the probability of micronutrient intake is 97.6% (lift value = 1.42)
4. If children living in Addis Ababa, children whose father completed higher education, and children aged 12–23 months, the probability of micronutrient intake is 96.9% (lift value = 1.4)
5. If children living in Gambella region, family wants no more children, and children born from mothers who had ANC follow-ups, the probability of micronutrient intake is 96.6% (lift value = 1.4)
6. If children living in Gambella region, children aged 12–23 months, and had a history of media exposure, the probability of being supplemented with micronutrient is 96.6% (lift value = 1.4)
7. If children living in Gambella region, children whose mother had work or occupation, and whose mothers gave birth at health facility, the probability of micronutrient intake is 95.4% (lift value = 1.37)

## Discussion

The aim of the study was to predict the micronutrient intake status among children aged 6–23 months in Ethiopia using advanced machine learning algorithms. Twelve different algorithms were tested, including Random Forest, Decision Tree, Naive Bayes, and others. All 12 algorithms used in the study performed well, with ROC values above the optimal threshold. The random forest classifier, catboost, and light gradient boosting classifier were particularly effective in identifying micronutrient intake status. Data balancing techniques were used to improve the accuracy of the final model. After balancing the data, the RF, Catboost, and LGB models performed the best overall. These findings are consistent with similar studies conducted in Rwanda (20) and Ethiopia (46), although the difference in data set sizes across the studies may have contributed to slight variations.

Analyzing the mean SHAP value report and waterfall plot provided valuable insights on factors influencing the prediction of micronutrient intake status among children aged 6–23 months. Factors like ANC, region, child age, and wealth index were found to be significant and influential. However, PNC utilization, father education, and desire more children had minimal impact on the classification outcome. Understanding these features can guide targeted interventions and policy decisions, improving the health and nutritional status of children in Ethiopia. These findings validate existing knowledge and evaluate the model’s effectiveness for more accurate interventions.

The other aim of the study was to identify the key predictors of micronutrient intake in children aged 6–23 months using the Boruta algorithm. Out of 23 features considered, 12 were found to be important for predicting micronutrient intake status. The Boruta algorithm revealed that machine learning models can uncover new variables and insights that traditional regression models may miss, providing valuable information for policy decisions.

The other objective of the study was to use association rule mining with the *a priori* algorithm to identify patterns and associations between independent predictors and the outcome variable. The top seven rules generated by the best model revealed that there was a 98.7% probability of micronutrient intake among children living in the Benishangul Gumuz region and coming from medium wealth index households is a striking observation. This result is supported with a study conducted in Ethiopia (47), Nigeria (48) and Bangladesh (49). This is the fact that household with better wealth may improve the nutritional intake for their children and Benishangul Gumuz region has agrarians’ community and they have access to serve diversified nutrient for their children. This finding suggests a strong association between the geographic region, economic status, and the likelihood of adequate micronutrient intake.

The model showed that the probability of having adequate micronutrient intake among a child living in the Gambella region, being from a rich household, and having a mother who gave birth at a health facility is claimed to be 97.8%. This result is supported by a study from recent EDHS (2016) (14). The possible justification is that, since agriculture is common in Gambella region, caregivers could get wild fruit and fish, which are good sources of micronutrients and Children from rich households, might have better access to a diverse and nutrient-rich diet, nutritional supplements, and healthcare resources. This economic advantage can contribute to a higher probability of meeting micronutrient requirements (50). Children born to mothers who deliver at health facilities may receive better post-natal care, including nutritional guidance and support. This could positively impact the child’s early development and micronutrient intake.

The reported probability of micronutrient intake is a substantial 96.9%, accompanied by a lift value of 1.4 for children living in Addis Ababa, children whose father completed higher education, and children aged 12–23 months. The result is in agreement with the studies conducted in east Africa, sub-Saharan Africa, and Nepal (15, 51, 52). This is due to the fact that higher education levels often correlate with increased awareness of nutrition and health, potentially impacting feeding practices, dietary choices, and overall child care. The higher the probability of obtaining micronutrient intake in 12–23 months could be explained by the fact that, at this age group, they could have better dietary diversity as they can eat family meals for themselves, and good complementary feeding practices are more common in urban than rural areas (14). Moreover, the late introduction of complementary foods and mothers’ and caregivers’ perceptions toward feeding diversified foods may contribute to lower consumption of micronutrients in lower age groups.

The Advanced ML result suggests that families in the Gambella region who express a desire for no more children are associated with a 95.4% probability of their children having adequate micronutrient intake. Studies support the idea that family size significantly influences the micronutrient intake of children (15, 53). This finding underscores the importance of family planning in contributing to better child nutrition outcomes. This is due to the fact that families with fewer children may have more resources available per child, enabling better access to nutritious food and healthcare. Alternatively, families with no desire for more children might be more focused on the well-being of their existing children. The result also indicates a positive association between micronutrient intake and mothers who had ANC follow-ups during pregnancy. This aligns with existing knowledge that adequate antenatal care is crucial for monitoring the health of both the mother and the developing child (14, 15). The positive correlation may be attributed to the health education and nutritional guidance provided during ANC visits. Mothers who attend ANC may receive information on proper nutrition during pregnancy and infancy, contributing to better nutritional practices. The result implies that implementing integrated healthcare approaches that combine family planning services with maternal and child health programs may further enhance the positive impact on micronutrient intake.

The advanced machine learning algorithms result shows that there is strong correlation between media exposure and micronutrient intake of children. The result is supported by studies conducted in Ethiopia and India (42, 43). This could be due to media may influence parental behavior, impacting their decision-making regarding child nutrition and encouraging them to prioritize their children’s nutritional needs.

## Strength and limitations of the study

This study had strengths in thoroughly evaluating 12 advanced machine learning algorithms and optimizing their performance through experimentation with data balancing and hyper parameter tuning. It also provided valuable insights into factors influencing micronutrient intake for targeted interventions and policies. However, limitations included reliance on existing data with potential limitations and biases, the inability to establish causal relationships or account for temporal changes, and the need for further validation in real-world settings with diverse populations to ensure reliability and generalizability.

## Conclusion and implication of the study

The study shows that machine learning can accurately predict micronutrient intake status among children in Ethiopia. All 12 algorithms performed well, with the random forest, catboost and LGB classifier being the most effective. These findings have important implications for targeted interventions and public health strategies. These findings carry significant implications for public health interventions in Ethiopia, as ML algorithms can be utilized to develop targeted strategies that promote the adoption of micronutrient intake.

The study identified several important risk factors for micronutrient intake among children aged 6–23 months. Advanced ML techniques, such as SHAP value logit coefficients, were used to overcome limitations of traditional ML approaches. The developed ML model, particularly the random forest, catboost, and LGB algorithm, is valuable for informing policies and interventions to prevent and minimize the burden of MN deficiency among children aged 6–23 months.

These identified risk factors can guide policymakers and healthcare providers in designing targeted interventions for different subgroups, improving the health and nutritional status of children and mitigating the impact of MN deficiency in resource-limited areas. However, further research is necessary to translate these findings into practical applications.

## Data availability statement

The datasets presented in this study can be found in online repositories. The names of the repository/repositories and accession number(s) can be found at: www.dhsprogram.com.

## Ethics statement

The studies involving humans were approved by central statistical agency-DHS program. The studies were conducted in accordance with the local legislation and institutional requirements. Written informed consent for participation in this study was provided by the participants’ legal guardians/next of kin.

## Author contributions

AZ: Conceptualization, Data curation, Formal analysis, Investigation, Methodology, Software, Validation, Visualization, Writing – original draft, Writing – review & editing. MAA: Writing – original draft, Writing – review & editing. AH: Writing – original draft, Writing – review & editing. BA: Writing – original draft, Writing – review & editing. MB: Writing – original draft, Writing – review & editing. WW: Writing – original draft, Writing – review & editing. AA: Writing – original draft, Writing – review & editing. HN: Formal analysis, Writing – original draft, Writing – review & editing.

## Footnotes

^1^ http://www.dhsprogram.com
