# Supplementary material for: Employing advanced supervised machine learning approaches for predicting micronutrient intake status among children aged 6–23 months in Ethiopia
Source: Front Nutr. 2024 Jun 11;11:1397399. doi: 10.3389/fnut.2024.1397399 (PMC11198118; doi:10.3389/fnut.2024.1397399)
Supplement: Supplementary file 1 [file Image_1.pdf]

A

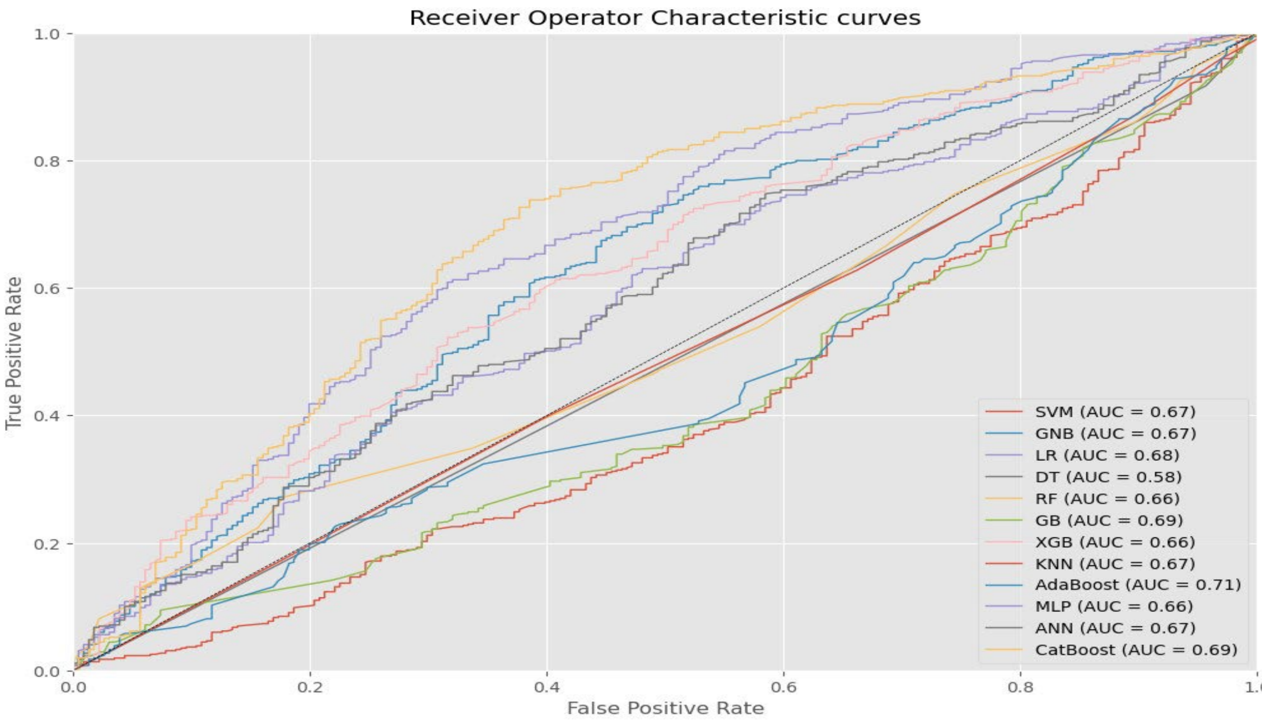
Receiver Operator Characteristic curves
True Positive Rate
False Positive Rate
0.0
0.2
0.4
0.6
0.8
1.0
SVM (AUC = 0.67)
GNB (AUC = 0.67)
LR (AUC = 0.68)
DT (AUC = 0.58)
RF (AUC = 0.66)
GB (AUC = 0.69)
XGB (AUC = 0.66)
KNN (AUC = 0.67)
AdaBoost (AUC = 0.71)
MLP (AUC = 0.66)
ANN (AUC = 0.67)
CatBoost (AUC = 0.69)

B

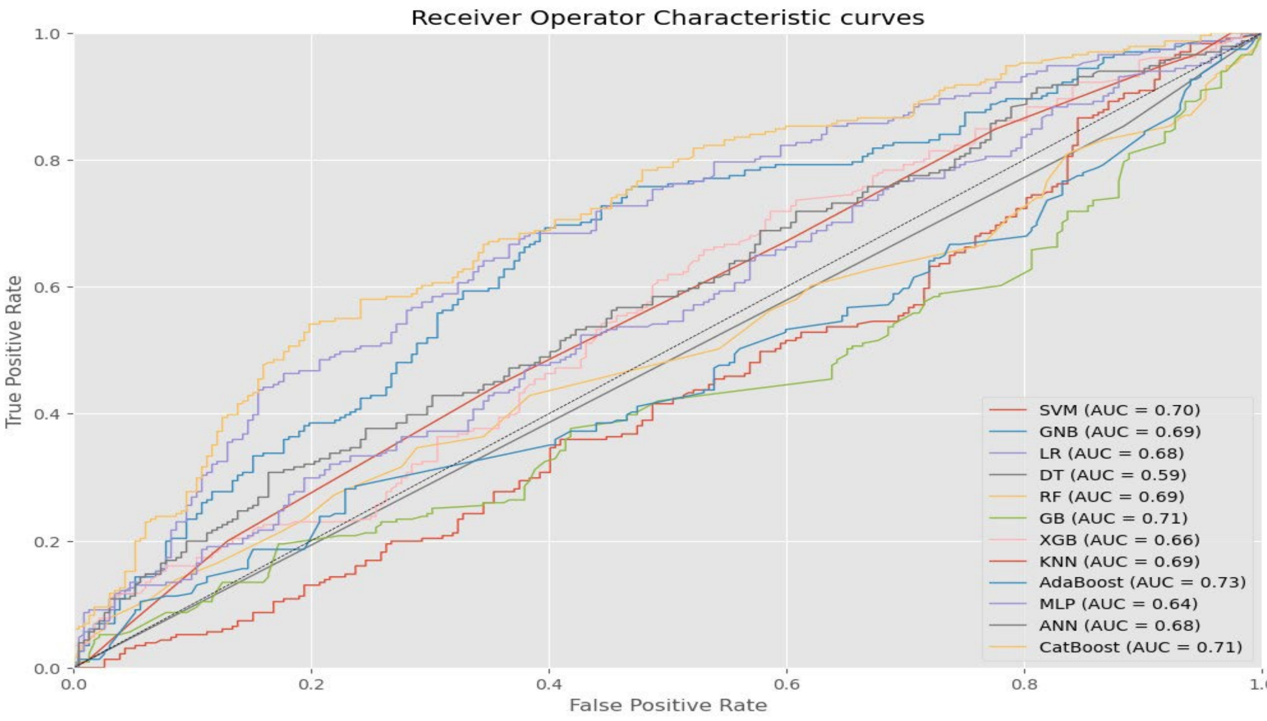
Receiver Operator Characteristic curves
True Positive Rate
False Positive Rate
0.0
0.2
0.4
0.6
0.8
1.0
SVM (AUC = 0.70)
GNB (AUC = 0.69)
LR (AUC = 0.68)
DT (AUC = 0.59)
RF (AUC = 0.69)
GB (AUC = 0.71)
XGB (AUC = 0.66)
KNN (AUC = 0.69)
AdaBoost (AUC = 0.73)
MLP (AUC = 0.64)
ANN (AUC = 0.68)
CatBoost (AUC = 0.71)

C

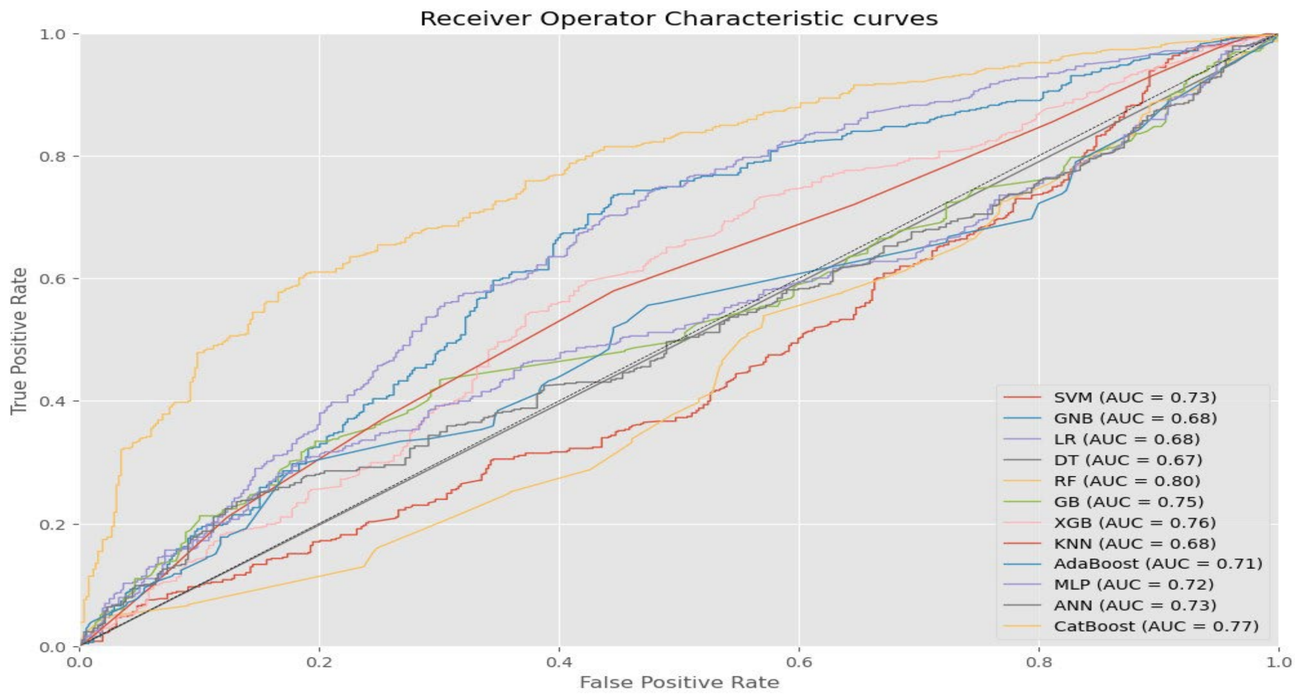
Receiver Operator Characteristic curves
True Positive Rate
False Positive Rate
0.0
0.2
0.4
0.6
0.8
1.0
SVM (AUC = 0.73)
GNB (AUC = 0.68)
LR (AUC = 0.68)
DT (AUC = 0.67)
RF (AUC = 0.80)
GB (AUC = 0.75)
XGB (AUC = 0.76)
KNN (AUC = 0.68)
AdaBoost (AUC = 0.71)
MLP (AUC = 0.72)
ANN (AUC = 0.73)
CatBoost (AUC = 0.77)

D

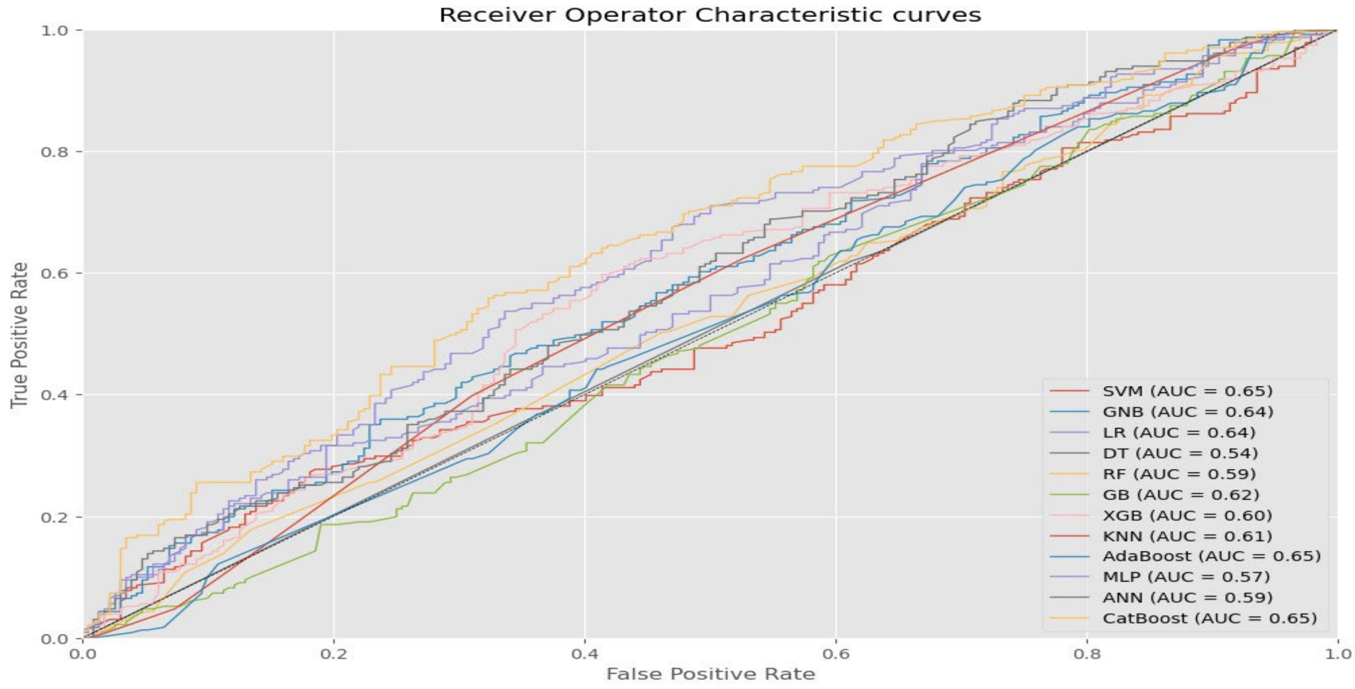
Receiver Operator Characteristic curves
True Positive Rate
False Positive Rate
0.0
0.2
0.4
0.6
0.8
1.0
SVM (AUC = 0.65)
GNB (AUC = 0.64)
LR (AUC = 0.64)
DT (AUC = 0.54)
RF (AUC = 0.59)
GB (AUC = 0.62)
XGB (AUC = 0.60)
KNN (AUC = 0.61)
AdaBoost (AUC = 0.65)
MLP (AUC = 0.57)
ANN (AUC = 0.59)
CatBoost (AUC = 0.65)

E

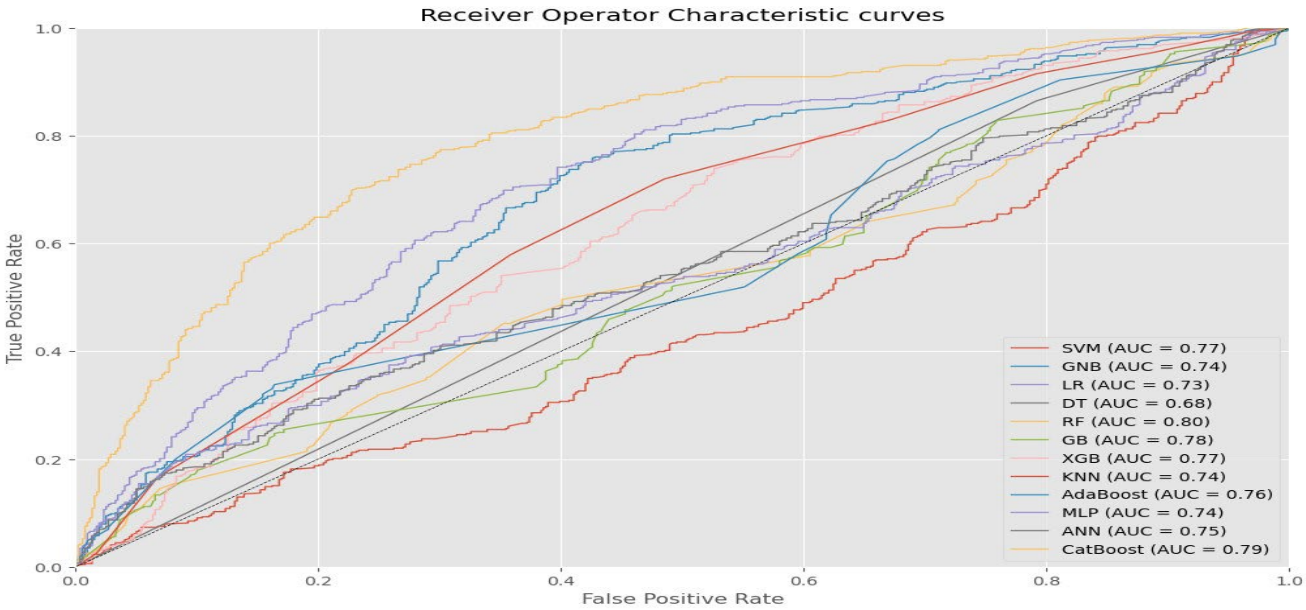

Receiver Operator Characteristic curves
True Positive Rate
False Positive Rate
0.0
0.2
0.4
0.6
0.8
1.0
SVM (AUC = 0.77)
GNB (AUC = 0.74)
LR (AUC = 0.73)
DT (AUC = 0.68)
RF (AUC = 0.80)
GB (AUC = 0.78)
XGB (AUC = 0.77)
KNN (AUC = 0.74)
AdaBoost (AUC = 0.76)
MLP (AUC = 0.74)
ANN (AUC = 0.75)
CatBoost (AUC = 0.79)

F

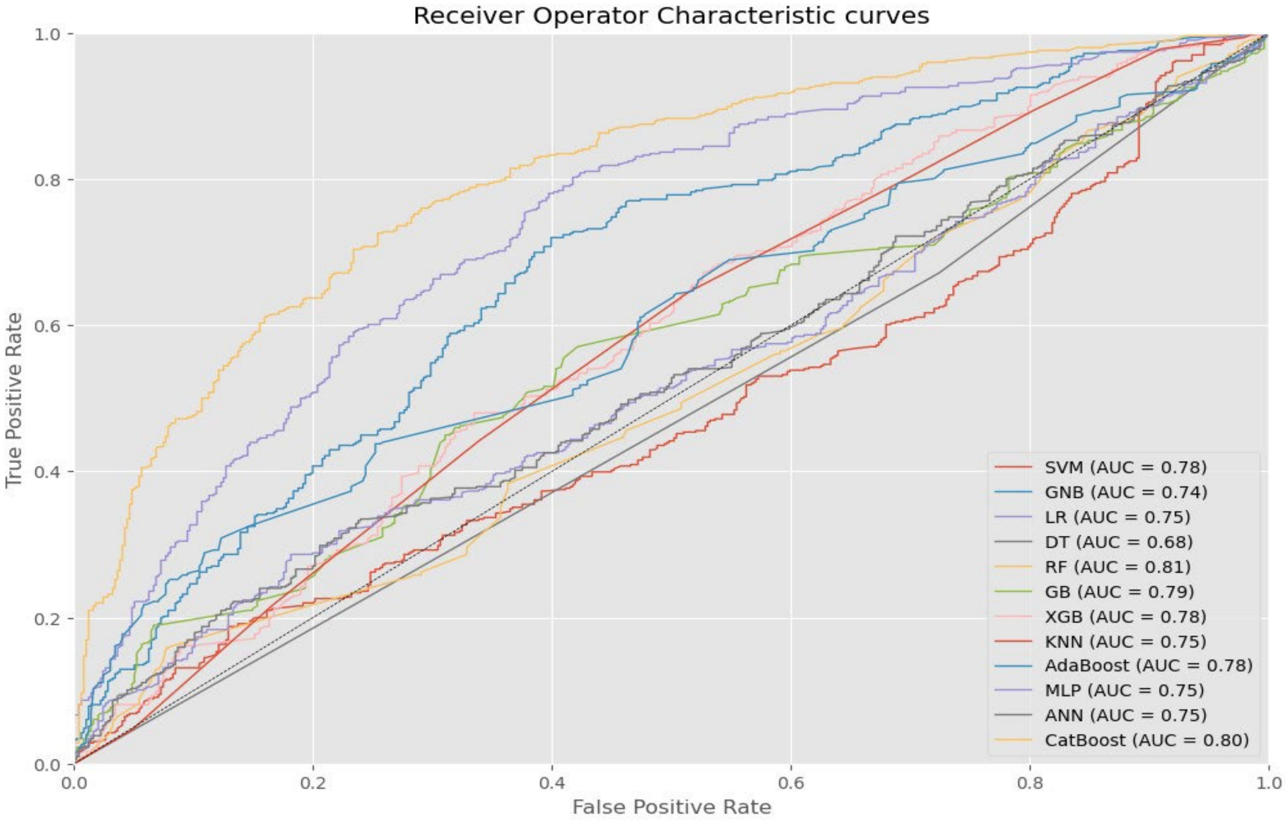

Receiver Operator Characteristic curves
True Positive Rate
False Positive Rate
0.0
0.2
0.4
0.6
0.8
1.0
SVM (AUC = 0.78)
GNB (AUC = 0.74)
LR (AUC = 0.75)
DT (AUC = 0.68)
RF (AUC = 0.81)
GB (AUC = 0.79)
XGB (AUC = 0.78)
KNN (AUC = 0.75)
AdaBoost (AUC = 0.78)
MLP (AUC = 0.75)
ANN (AUC = 0.75)
CatBoost (AUC = 0.80)

G

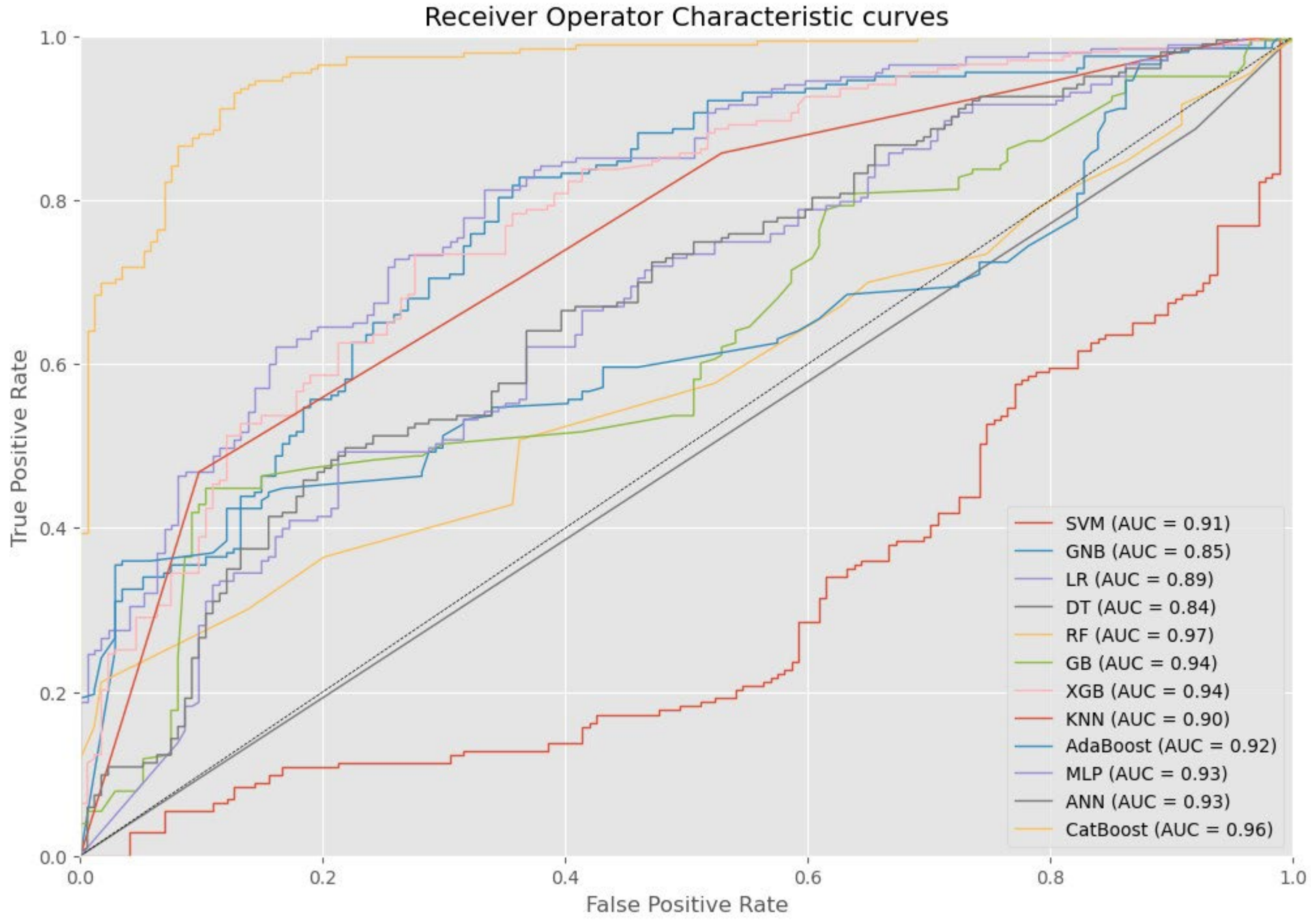

Figure-1: AUC value of twelve ML algorithm based on A) unbalanced data, B) under sampling, C) over sampling, D) Near miss, E) SMOTE, F) SMOTE Tomek, and G) SMOTE ENN data balancing techniques
