# Supplementary material for: Employing advanced supervised machine learning approaches for predicting micronutrient intake status among children aged 6–23 months in Ethiopia
Source: Front Nutr. 2024 Jun 11;11:1397399. doi: 10.3389/fnut.2024.1397399 (PMC11198118; doi:10.3389/fnut.2024.1397399)
Supplement: Supplementary file 2 [file Image_2.pdf]

A

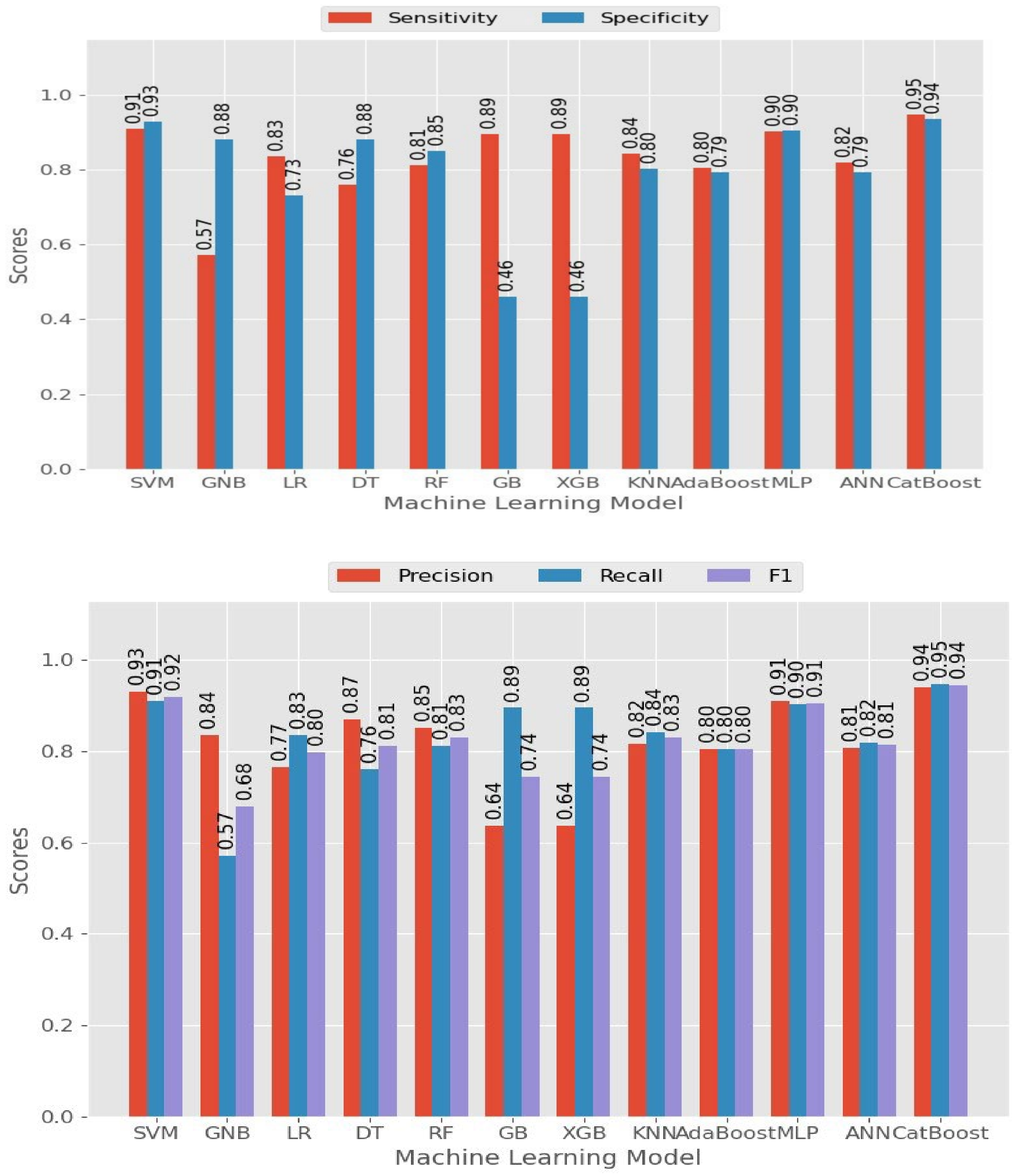

Sensitivity
Specificity
Scores
1.0
0.8
0.6
0.4
0.2
0.0
0.91 0.93
0.57 0.88
0.83 0.73
0.76 0.88
0.81 0.85
0.89 0.46
0.89 0.46
0.84 0.80
0.80 0.79
0.90 0.90
0.82 0.79
0.95 0.94
SVM GNB LR DT RF GB XGB KNN AdaBoost MLP ANN CatBoost
Machine Learning Model
Precision
Recall
F1
Scores
1.0
0.8
0.6
0.4
0.2
0.0
0.93 0.91 0.92
0.84 0.57 0.68
0.77 0.83 0.80
0.87 0.76 0.81
0.85 0.81 0.83
0.64 0.89 0.74
0.64 0.89 0.74
0.82 0.84 0.83
0.80 0.80 0.80
0.91 0.90 0.91
0.81 0.82 0.81
0.94 0.95 0.94
SVM GNB LR DT RF GB XGB KNN AdaBoost MLP ANN CatBoost
Machine Learning Model

**B**

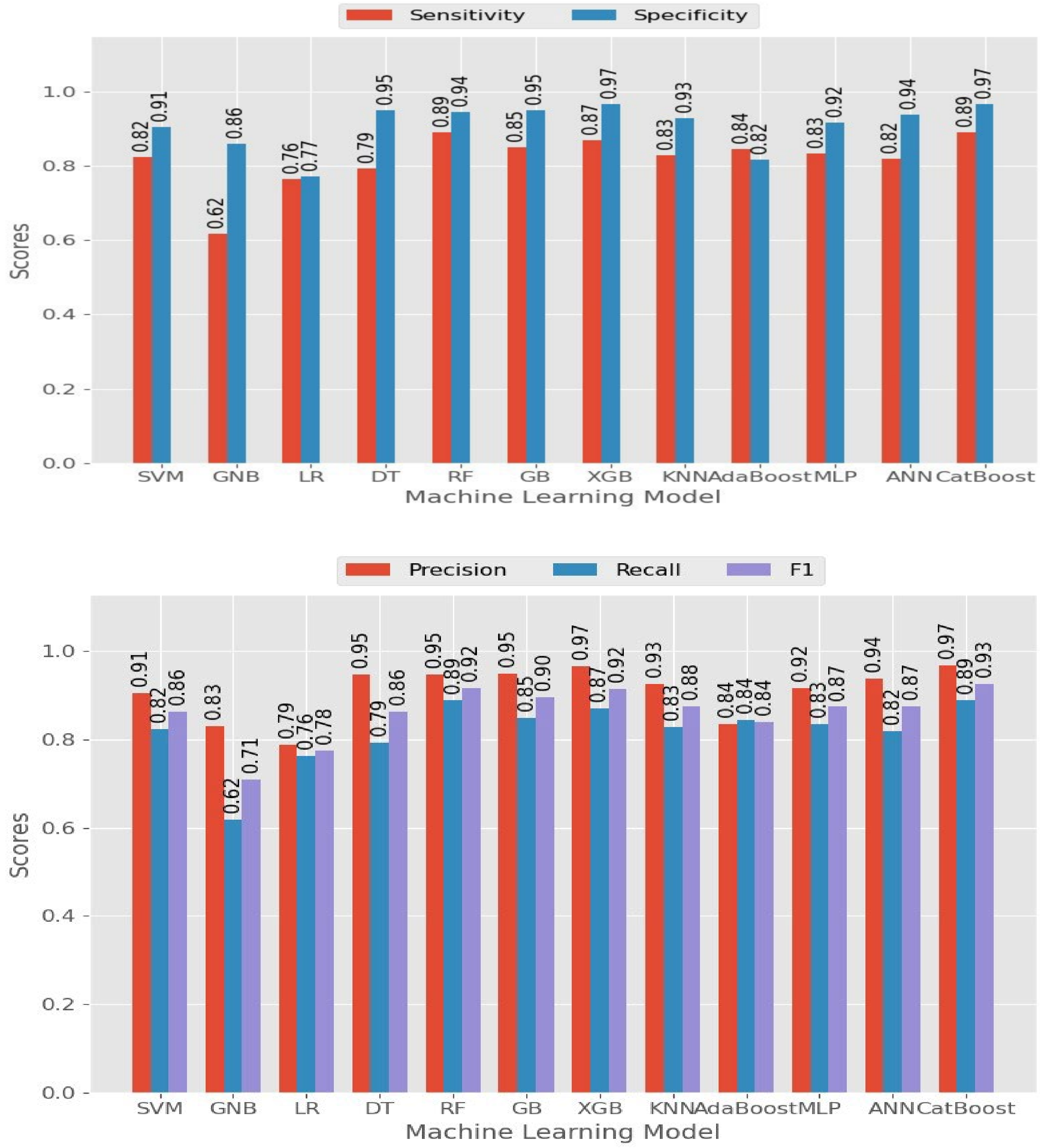

Sensitivity
Specificity
Scores
Machine Learning Model
SVM
GNB
LR
DT
RF
GB
XGB
KNN
AdaBoost
MLP
ANN
CatBoost
Precision
Recall
F1
Scores
Machine Learning Model

C

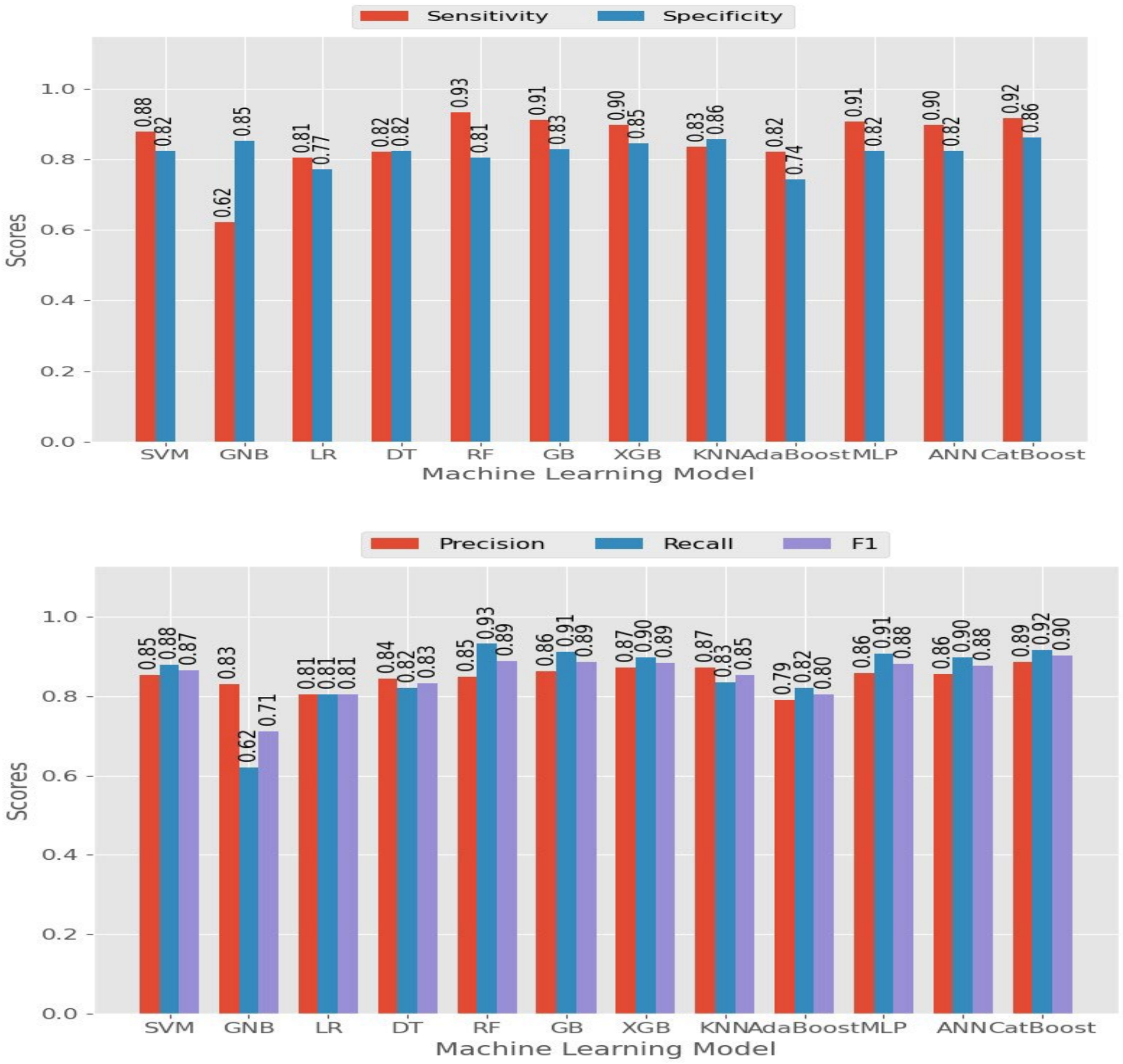


Figure-2: Evaluation metrics value of the twelve algorithms based on A) Bayesian tuning, B) Grid search, and C) random search hyper parameter tuning techniques
